# Risk of Instrumental Delivery in Maternal Obesity: Estimates With Measures of Effect Size

**DOI:** 10.31486/toj.24.0041

**Published:** 2024

**Authors:** Melinda Chai, Amanda Vining, Joseph Koveleskie, William Sumrall, Bobby D. Nossaman

**Affiliations:** ^1^ The University of Queensland Medical School, Ochsner Clinical School, New Orleans, LA; ^2^ Department of Anesthesiology and Perioperative Medicine, Ochsner Clinic Foundation, New Orleans, LA

**Keywords:** *Comorbidity*, *delivery–obstetric*, *obesity–maternal*, *surgical instruments*

## Abstract

**Background:** Obesity, defined as a body mass index ≥30 kg/m^2^, is epidemic in the United States and is associated with increased risks of adverse events. Studies have examined the role of maternal obesity on the incidence of instrumental vaginal delivery, but the results are divided. However, these analyses used frequentist tests that risk false discovery. The purpose of this retrospective study was to quantify the association of maternal obesity to the need for instrumental delivery with measures of effect size. Measures of effect size allow assessment of the impact of clinical risk factors on outcomes of interest.

**Methods:** All parturients aged ≥18 years in active labor at our facility from January 2018 to May 2019 were entered into this study. Patient demographics, previously reported comorbidities, and obstetric parameters were collected and analyzed to determine the clinical impact of maternal obesity on the incidence of instrumental delivery. One effect size measure, risk differences, was used to quantify the clinical effect of maternal obesity on the need for instrumental delivery. A generalized linear model was used to standardize the measures of effect size of previously reported comorbidities, including maternal obesity, and to determine their association with the need for instrumental delivery.

**Results:** The incidences of chronic and gestational hypertension, preeclampsia, chronic diabetes, and reactive airway disease were higher in parturients with maternal obesity. Risk differences due to maternal obesity were observed in parturients presenting with shoulder dystocia but not in those who underwent oxytocin induction or in nulliparous parturients. Following regression analysis, maternal obesity did not clinically impact the need for instrumental delivery.

**Conclusion:** These findings suggest that maternal obesity did not have a clinical impact on the need for instrumental delivery.

## INTRODUCTION

Obesity, defined by the World Health Organization as a body mass index (BMI) ≥30 kg/m^2^, is epidemic in the United States and is associated with increased maternal risks.^1-13^ Studies have shown that maternal obesity increases the risk of gestational diabetes, pregnancy-induced hypertension, preeclampsia, shoulder dystocia, and the need for labor induction.^1-13^ Studies have also examined the role of maternal obesity (BMI ≥30 kg/m^2^) on the incidence of instrumental delivery, but these results are divided.^1-13^ However, these analyses used frequentist tests in which statistical significance may be attributable to high sample sizes that risk high false discovery rates,^14^ or the results were expressed with odds ratios when event rates were common.^15-17^ The purpose of this observational study was to quantify the magnitude and precision of the association of maternal obesity to the need for instrumental delivery.

## METHODS

Following institutional board approval, all parturients aged ≥18 years in active labor from January 2018 to May 2019 at Ochsner Health in New Orleans were entered into this study. Patient demographics, previously reported comorbidities, and obstetric parameters were collected to examine the role of maternal obesity on the mode of delivery (instrumental vaginal delivery vs normal spontaneous vaginal delivery) in parturients during active labor.

### Statistics

Categorical variables are presented as counts and percentages with confidence intervals (CI), and differences between BMI groups (BMI ≥30 kg/m^2^ vs BMI <30 kg/m^2^) were assessed with chi-square tests. Continuous variables with skewed distributions are presented as medians with 25% to 75% interquartile ranges [IQR], and differences between BMI groups were assessed with the Wilcoxon rank-sum test. Risk differences with 95% CIs were used as measures of effect size to assess the magnitude and precision of individual previously reported comorbidities for the outcome of interest, the need for instrumental delivery.^18-23^ When the ranges of these CIs do not contain zero, a clinical impact is suggested.

A generalized linear model^24^ was constructed to quantify the association of adjusted risk differences of previously reported comorbidities to the need for instrumental delivery. Clinical impacts of the previously reported comorbidities in this model are expressed as standardized risk differences with 95% confidence limits (CL).^24^ Frequentist tests are shown for comparison, with *P* values set for significance at <0.005 to minimize the risk of false discovery rates or of declaring associations significant by chance alone.^14,25,26^ The statistical program JMP Pro 17.2 (SAS Institute Inc) was used for this study.

## RESULTS

Demographics, comorbidities, and measured obstetric parameters for 1,183 parturients undergoing active labor are shown in Table 1. When delivery BMI was partitioned into obese (BMI ≥30 kg/m^2^) and nonobese (BMI <30 kg/m^2^) groups, 592/1,183 or 50.0% (CI 47.2%-52.9%) of the parturients were observed to be obese. Risk differences were observed in the obese parturients with histories of chronic hypertension, gestational hypertension, preeclampsia, chronic diabetes, and reactive airway disease. Risk differences due to maternal obesity were observed in parturients presenting with shoulder dystocia, but not for those who underwent oxytocin induction or for nulliparous parturients. No risk differences were observed between the 2 BMI groups by the type of occiput presentation or in the mode of delivery.

**Table 1.** Demographics, Comorbidities, and Obstetric Risk Factors of Parturients by Body Mass Index (BMI), n=1,183

Variable	BMI ≥30 kg/m^2^, n=592	BMI <30 kg/m^2^, n=591	*P* Value
Demographics, median [IQR]			
Age, years	29 [26-33]	30 [27-33]	0.0470
Pregravid weight, kg	80 [69-92]	60 [55-65]	<0.0001
Delivery weight, kg	93 [83-104]	72 [66-77]	<0.0001
Percent change in weight	15 [11-20]	17 [13-19]	0.0684
Delivery BMI, kg/m^2^	35 [32-38]	27 [25-28]	<0.0001
			**Risk Difference, CI**
Comorbidities			
Chronic hypertension	28 (4.7)	10 (1.7)	**0.03, 0.01 to 0.05**
Gestational hypertension	90 (15.2)	46 (7.8)	**0.07, 0.04 to 0.11**
Preeclampsia	70 (11.8)	36 (6.1)	**0.06, 0.02 to 0.09**
Chronic diabetes	15 (2.5)	2 (0.3)	**0.02, 0.008 to 0.04**
Gestational diabetes	37 (6.2)	24 (4.1)	0.02, –0.003 to 0.05
Reactive airway disease	71 (12.0)	41 (6.9)	**0.05, 0.02 to 0.08**
History of drug abuse	7 (1.2)	10 (1.7)	–0.005, –0.02 to 0.008
History of tobacco abuse	15 (2.5)	14 (2.4)	0.002, –0.02 to 0.02
Obstetric parameters			
Shoulder dystocia	62 (10.5)	20 (3.4)	**0.07, 0.04 to 0.1**
Oxytocin induction	469 (79.2)	442 (74.8)	0.04, –0.004 to 0.09
Nulliparity	229 (38.7)	243 (41.1)	–0.02, –0.08 to 0.03
Fetal presentation			
Occiput anterior (OA) including left OA and right OA	549 (92.7)	558 (94.4)	–0.02, –0.05 to 0.01
Occiput posterior (OP) including left OP and right OP	36 (6.1)	23 (3.9)	0.02, –0.003 to 0.05
Occiput transverse	7 (1.2)	7 (1.2)	–0.00002, –0.01 to 0.01
Mode of delivery			
Instrumental	51 (8.6)	50 (8.5)	0.001, –0.03 to 0.03
Normal spontaneous vaginal	541 (91.4)	540 (91.4)	

Notes: Data are presented as n (%) unless otherwise indicated. Risk differences due to maternal obesity are in bold. CI, 95% confidence interval. IQR, 25%-75% interquartile range.

Admitting diagnoses by BMI group are shown in Table 2. Risk differences due to obesity were only observed in parturients scheduled for labor induction or who presented with premature rupture of membranes.

**Table 2.** Admitting Diagnoses of Parturients by Body Mass Index (BMI), n=1,183

Admitting Diagnosis	BMI ≥30 kg/m^2^, n=592	BMI <30 kg/m^2^, n=591	Risk Difference, CI
Encounter for trial of labor	7 (1.2)	8 (1.4)	–0.002, –0.016 to 0.012
Scheduled labor induction	336 (56.8)	268 (45.3)	**0.11, 0.057 to 0.17**
Uterine contractions	160 (27.0)	188 (31.8)	–0.05, –0.10 to 0.002
Rupture of membranes	44 (7.4)	46 (7.8)	–0.004, –0.03 to 0.03
Premature rupture of membranes	29 (4.9)	54 (9.1)	**–0.04, –0.07 to –0.01**
Preterm premature rupture of membranes	11 (1.9)	19 (3.2)	–0.01, –0.03 to 0.007
Preterm labor	6 (1.0)	6 (1.0)	–0.0001, –0.013 to 0.013

Notes: Data are presented as n (%) unless otherwise indicated. Risk differences due to maternal obesity are in bold. Whole model chi-square=20.0, *P*=0.0028.

Eighty-two cases of shoulder dystocia were observed in this study. The clinical impact of maternal obesity on the association of shoulder dystocia to the mode of delivery is shown in Table 3(A). Risk differences were calculated, and no clinical impact of obesity was observed in parturients receiving instrumental delivery.

**Table 3.** Clinical Impact of Maternal Obesity on the Association of (A) Shoulder Dystocia, (B) Oxytocin Use, and (C) Nulliparity to the Mode of Delivery

**(A) Presence of Shoulder Dystocia, n=1,176**				
	**Instrumental Delivery**			
	**Yes**	**No**	**Risk Difference, CI**	**χ^2^, *P* Value**
BMI ≥30 kg/m^2^	8 (16)	43 (84)	0.10, –0.03 to 0.21	2.4, 0.1182
BMI <30 kg/m^2^	3 (6)	47 (94)		
	**NSV Delivery**			
BMI ≥30 kg/m^2^	54 (10)	486 (90)	0.07, 0.04 to 0.10	20.3, <0.0001
BMI <30 kg/m^2^	17 (3)	518 (97)		
**(B) Oxytocin Use, n=1,182**				
	**Instrumental Delivery**			
	**Yes**	**No**	**Risk Difference, CI**	**χ^2^, *P* Value**
BMI ≥30 kg/m^2^	35 (69)	16 (31)	–0.15, –0.31 to 0.01	3.3, 0.0696
BMI <30 kg/m^2^	42 (84)	8 (16)		
	**NSV Delivery**			
BMI ≥30 kg/m^2^	434 (80)	107 (20)	0.06, 0.01 to 0.11	5.8, 0.0161
BMI <30 kg/m^2^	400 (74)	140 (26)		
**(C) Nulliparous Parturient, n=1,182**				
	**Instrumental Delivery**			
	**Yes**	**No**	**Risk Difference, CI**	**χ^2^, *P* Value**
BMI ≥30 kg/m^2^	28 (55)	23 (45)	0.03, –0.16 to 0.22	0.9, 0.7700
BMI <30 kg/m^2^	26 (52)	24 (48)		
	**NSV Delivery**			
BMI ≥30 kg/m^2^	201 (37)	340 (63)	–0.03, –0.09 to 0.03	1.0, 0.3061
BMI <30 kg/m^2^	217 (40)	323 (60)		

Notes: Data are presented as n (%) unless otherwise indicated. Percentages are calculated across rows. CI, 95% confidence interval. χ^2^, chi-square statistic. *P* values <0.005 are statistically significant.^26^

BMI, body mass index; NSV, normal spontaneous vaginal.

Seventy-seven percent of patients (CI 75%-79%) received oxytocin, either with scheduled admission or initiated during labor. The clinical impact of maternal obesity on the association of oxytocin use to mode of delivery is shown in Table 3(B). No clinical risk differences in the need for instrumental delivery were observed in obese parturients compared to nonobese parturients.

Risk differences were calculated for the role of maternal obesity on the association of nulliparity to the mode of delivery, and the results of this association are shown in Table 3(C). No clinical risk differences of maternal obesity were observed with the association of nulliparity to the mode of delivery.

Risk differences were standardized for the previously reported comorbidities associated with maternal obesity, and the results of this generalized linear regression analysis are shown in Table 4. None of the comorbidities—including maternal obesity—was associated with the need for instrumental delivery.

**Table 4.** Adjusted Risk Differences of Previously Reported Comorbidities and the Risk of Instrumental Delivery

Term	Standardized Risk Difference	95% CL	Std Error	Log-Ratio Chi-Square	*P* Value
Intercept	0.14	0.055 to 0.253	0.050	7.5	0.0062
BMI ≥30 kg/m^2^	–0.002	–0.019 to 0.014	0.008	0.1	0.7694
Chronic hypertension	–0.021	–0.042 to 0.029	0.018	0.9	0.3341
Gestational hypertension	0.004	–0.018 to 0.033	0.013	0.1	0.7557
Preeclampsia	0.030	–0.001 to 0.068	0.018	3.7	0.0549
Chronic diabetes	0.010	–0.037 to 0.111	0.038	0.1	0.7754
Gestational diabetes	0.026	–0.010 to 0.076	0.022	1.8	0.1789
Reactive airway disease	0.011	–0.015 to 0.045	0.015	0.6	0.4314

Notes: Whole model chi-square=7.4, degrees of freedom=8, *P*=0.3922. Goodness of fit statistic: log-ratio chi-square=1182, *P*=0.4292. *P* values <0.005 are statistically significant.^26^

BMI, body mass index; CL, confidence limit; Std Error, standard error of the risk difference.

## DISCUSSION

In this study of 1,183 parturients, the incidence of maternal obesity was 50%. A clinical impact of obesity was suggested in parturients with histories of chronic and gestational hypertension, preeclampsia, chronic diabetes, and reactive airway disease but not in obese parturients with histories of gestational diabetes or of drug and tobacco abuse. These findings are comparable to observations reported in other studies.^1-13^

### Shoulder Dystocia and Maternal Obesity

Although a higher incidence of parturients presenting with shoulder dystocia was observed in the obese group, the incidence of instrumental delivery in shoulder dystocia cases was the same for both BMI groups, as were the incidences of instrumental delivery in those who underwent oxytocin induction and in nulliparous parturients. Although Scott-Pillai et al^2^ and Robinson et al^11^ observed higher associations of shoulder dystocia with increasing obesity, both groups reported a lower need for instrumental delivery in their obese parturients. Although Usha Kiran and colleagues^12^ reported a very low incidence of shoulder dystocia, they observed no differences in the need for assisted vaginal delivery in their obese parturients.

### Instrumental Delivery and Maternal Obesity

Studies have also examined the role of maternal obesity on the incidence of instrumental delivery, but these results were divided. Blomberg,^13^ Cedergren,^6,7^ Nohr et al,^8^ Schrauwers and Dekker,^9^ and Sydsjö et al^10^ reported higher associations of instrumental delivery with maternal obesity. In contrast, Pettersen-Dahl et al,^1^ Scott-Pillai et al,^2^ Foo et al,^3^ Roman et al,^4^ Robinson et al,^11^ and Sarkar et al^5^ did not observe an increased association of instrumental delivery with maternal obesity. The reasons for these decreasing associations are not clear, but Pettersen-Dahl et al suggested that the obstetricians may have decided to perform early cesarean deliveries rather than proceed with instrumental delivery.^1^ Scott-Pillai et al also observed increased rates of cesarean delivery, whether elective or emergency, in their obese parturients with corresponding lower rates of normal or instrumental deliveries.^2^ Finally, Roman et al observed an association of cesarean delivery with shoulder dystocia and a resultant lower need for instrumental delivery in their obese parturient population.^4^

### Admitting Diagnoses and Maternal Obesity

The primary admitting diagnoses in this study were similar to those reported in other studies,^1-4^ except for a higher incidence of parturients admitted for scheduled oxytocin induction in this study. Pettersen-Dahl and colleagues reported admitting incidences for oxytocin induction of 47.3% for obese parturients and 17.1% for nonobese parturients.^1^ Jensen and colleagues reported admitting oxytocin induction incidences of 12.4% in their nonobese group and 14.1% and 23.4% in their 2 obese groups.^27^ These incidences^1,27^ are lower than our observations (Table 2), and the differences in admitting oxytocin induction incidences may reflect distinctions in practice patterns. However, the risk differences analysis in Table 3(B) shows that oxytocin use was not associated with the need for instrumental delivery.

### Previously Reported Comorbidities and Maternal Obesity

We performed generalized linear regression analysis on previously reported comorbidities, including maternal obesity, to standardize their interplay with the need for instrumental delivery. None of the comorbidities, including maternal obesity, was associated with the need for instrumental delivery. In the comparative studies,^1-13^ odds ratios were frequently used as a measure of effect size, but odds ratios have the potential to overexpress relative risk. Odds ratios calculated on proportions exceeding 20% will exaggerate the effect, especially when the proportion is large in one group compared to the proportion in the other group.^15-17^ Additionally, odds ratios cannot be calculated when no events are recorded in any comparative group. Finally, few clinicians are truly facile with this statistic; odds ratios do not account for the incidence of the outcome in question and do not translate well during patient discussions at the bedside,^28^ whereas measures of effect size, such as risk differences, can be clearly understood by physicians, patients, and their families.^15,28^

### Limitations and Strengths

A limitation of retrospective studies is the completeness of medical record data.^29^ However, a strength of this study is the near 100% data collection (1.1% missing data) because of the development of electronic medical records. Another limitation of this study is the lack of data on early cesarean deliveries because we did not study decisions for this surgical option.

A strength of this study is the use of measures of effect size. Health care professionals should consider integrating measures of effect size into their clinical evaluations to assess the clinical importance of their findings.^18,20-23^ Effect sizes provide insight into the numeric relationship between binary variables associated with an outcome of interest. This effect size is referred to as the risk difference. Incorporating these measures can enhance the depth of analysis and interpretation in clinical settings. When medical researchers only use frequentist tests in investigation of their findings, statistically significant results may or may not have clinical importance, and moreover, there is a 29.6% chance that their findings are not reproducable.^30^ An additional measure to improve the value of statistical significance not due to chance is to reset the *P* values of frequentist statistics to the now recommended value of <0.005,^26^ which reduces the potential rate of false discovery from 29.6% to <6.9%.^14^

Another limitation of this study is potential bias due to confounding. However, a robust statistical method was used to standardize the interplay of previously reported predictors against the outcome in question. Also, the magnitude and precision of a predictor of interest on the outcome can be realized. Generalized linear modeling provides a useful tool to standardize measures of effect size, as the standardization expresses information about the clinical magnitude and the precision of that magnitude of the association rather than the inferences based on frequentist testing.^31^

Measures of effect size provide robust clinical evidence in support of clinical decisions.^18,19^ Whether the effect size is clinically relevant depends upon the importance of the risk factor and the risk differences with the CI ranges of the 2 outcome groups. Finally, standardized risk differences hold an advantage over odds ratios as they express information about the occurrence of the outcome in addition to information about the magnitude and precision of the association.^31^

## CONCLUSION

Although risk differences for chronic hypertension, gestational hypertension, preeclampsia, chronic diabetes, and reactive airway disease were increased in parturients with maternal obesity, these parameters, once standardized, did not play a clinical role on the need for instrumental delivery. Although a higher incidence of parturients presented with shoulder dystocia in the obese group, the incidence of instrumental delivery was not clinically different in the nonobese and obese groups, nor was any difference seen in those who underwent oxytocin induction or in nulliparous parturients. Maternal obesity did not play a role in the need for instrumental delivery. The use of measures of effect size provides clinical assessments of outcomes rather than inferences based on statistical significance values obtained from frequentist statistics.

## Notes

*This article meets the Accreditation Council for Graduate Medical Education and the American Board of Medical Specialties Maintenance of Certification competencies for Patient Care and Medical Knowledge.*
